# Dissection of Estrogen Receptor Alpha Signaling Pathways in Osteoblasts Using RNA-Sequencing

**DOI:** 10.1371/journal.pone.0095987

**Published:** 2014-04-28

**Authors:** Matthew M. Roforth, Elizabeth J. Atkinson, Ellis R. Levin, Sundeep Khosla, David G. Monroe

**Affiliations:** 1 Endocrine Research Unit and Kogod Center on Aging, Mayo Clinic, Rochester, Minnesota, United States of America; 2 Endocrinology and Metabolism, VA Long Beach Healthcare System and University of California, Irvine, California, United States of Ameirca; II Università di Napoli, Italy

## Abstract

The effects of 17-β-estradiol in osteoblasts are primarily mediated by the nuclear transcription factors, estrogen receptor (ER)α and ERβ. ERs function through three general modes of action: DNA-binding dependent through estrogen response elements (EREs; designated nuclear ERE signaling); nuclear signaling via protein-protein interactions to other transcription factors (nuclear non-ERE signaling); and extra-nuclear signaling (membrane-bound functions of ERs). Identification of the specific transcriptional signatures regulated by each of these modes of action should contribute to an enhanced understanding of estrogen signaling in osteoblasts. To achieve this goal, we utilized specific mutations of ERα that eliminate the ability of the receptor to signal through a specific mode of action. The non-classical ERα knock-in (NERKI) mutation is incapable of signaling through direct DNA binding to EREs and the nuclear only ERα (NOER) mutation eliminates all membrane-localized signaling. Comparison of the gene expression patterns elicited by these mutations with the wild-type ERα (WT) pattern provides mode-specific data concerning transcriptional regulation by ERα. We expressed these constructs in the ER-negative osteoblastic cell line hFOB (−/+ estrogen) and performed global RNA-sequencing. Using a series of pair-wise comparisons, we generated three lists of genes that were regulated either by the nuclear ERE-dependent, nuclear ERE-independent, or extra-nuclear actions of ERα. Pathway and gene ontology analyses revealed that genes regulated through the nuclear ERE and nuclear non-ERE pathways were largely involved in transcriptional regulation, whereas genes regulated through extra-nuclear mechanisms are involved in cytoplasmic signaling transduction pathways. We also intersected our data with genes linked to bone density and fractures from a recent genome-wide association study and found 25 of 72 genes (35%) regulated by estrogen. These data provide a comprehensive list of genes and pathways targeted by these specific modes of ERα action and suggest that “mode-specific” ligands could be developed to modulate specific ERα functionality in bone.

## Introduction

Estrogen (E) signaling is mediated by two receptors, estrogen receptor (ER)α and ERβ. Although important for many tissue systems, ER signaling is of particular interest with regards to bone biology, as declining E levels during the menopause lead to increased bone turnover and bone loss, as is observed in postmenopausal osteoporosis [1]. Although extensive research has been conducted to understand the role of ERs in bone through use of cellular and animal models, the understanding of gene regulation and cellular pathways regulated by E in osteoblasts is still incomplete.

Mechanistically, ERs modulate gene expression using a number of distinct modes of action. The classical pathway involves direct binding to estrogen response elements (EREs) in the control regions of genes, whereas an alternative nuclear pathway involves protein-protein interactions which are ERE-independent. To facilitate study of these pathways, an ERα mutation [non-classical ERα knock-in (NERKI)] has been made that eliminates ERE but retains non-ERE signaling (both nuclear and extra-nuclear) [2], allowing investigators to study the effects of non-ERE mediated ERα signaling alone. Mice harboring this mutation display an osteoporotic phenotype, demonstrating that nuclear ERE-mediated signaling is important in regulating bone metabolism [3], [4], [5].

It has become increasingly appreciated that the nuclear actions of ERs cannot completely explain all aspects of ER signaling. Compelling evidence demonstrates that estrogen can act rapidly (within 5–15 minutes of E treatment) through non-genomic mechanisms, to regulate signal transduction through pathways such as ERK and PI3K [6], [7], [8], [9]. This mechanism involves tethering of ERα to the plasma membrane (PM) via palmitoylation of a cysteine residue in its E domain, which facilitates association with caveolin-1 [7]. A mutation of this cysteine has been made (termed “nuclear-only ER” or NOER) that completely eliminates membrane localization of ERα [10], and thereby prevents PM ERα signaling. Therefore, in comparison with wild-type ERα (WT), the NOER receptor can be used as a tool to identify those genes regulated through either the nuclear or membrane-associated ERα pathways.

In an effort to identify target genes and pathways regulated by each of these distinct signaling mechanisms, we expressed WT, NERKI, or NOER receptors in an ER-negative osteoblast cell model and measured global gene expression patterns following estrogen treatment using RNAseq. Comparison of estrogen-dependent gene expression patterns allowed us to compartmentalize these patterns according to the known cellular mechanisms used by ERα, such as dependence on an ERE or whether these genes are regulated through the extra-nuclear (e.g. membrane-associated) function of ERα. These data identify characteristic E-regulated gene signatures from three distinct ERα regulatory mechanisms, which could potentially be used for targeting specific cellular ERα pathways.

## Materials and Methods

### Cell culture reagents, adenoviruses and infection of hFOB 1.19 cells

**Figure 1:**
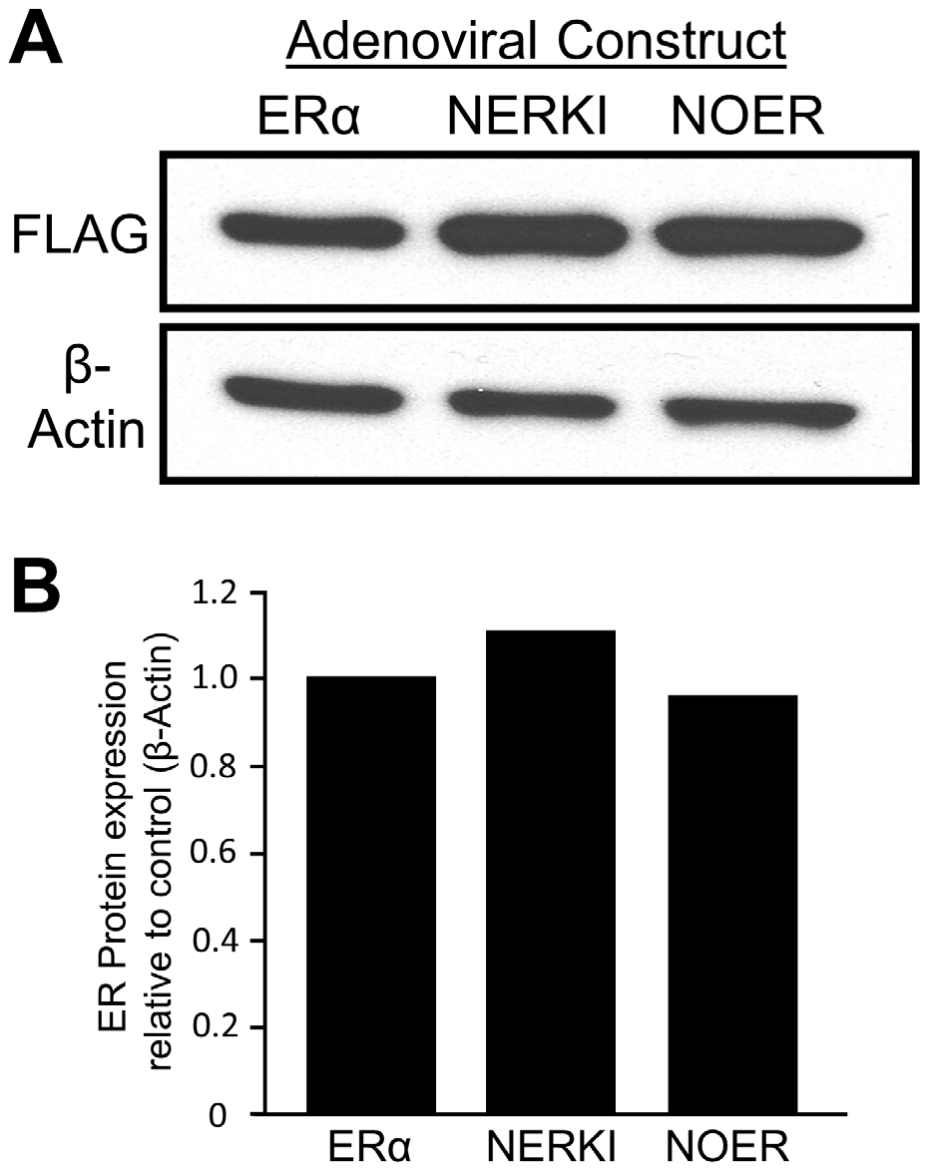
Estrogen receptor expression in hFOB cells. A) hFOB cells were infected with Ad-ERα, Ad-NERKI and Ad-NOER and cultured for 24 h. Protein extracts were prepared and a western blot was performed using the anti-FLAG and anti-β-actin antibodies. B) Densitometry was performed and the data are expressed as ER expression relative to the β-actin control.

The hFOB 1.19 human fetal osteoblastic cell line (hFOB), produced by Harris and colleagues [11], was passaged in phenol red-free αMEM growth medium (Invitrogen, Carlsbad, CA) supplemented with 1X antibiotic/antimycotic (Invitrogen), 10% (v/v) fetal bovine serum (Hyclone, Logan, UT) and 300 ug/mL G418 selection antibiotic (Invitrogen). The Ad-ERα (expressing WT mouse ERα) and Ad-NERKI (expressing mouse ERα containing the double mutation E207A/G208A, which is incapable of binding DNA [2]) adenoviral vectors were produced as previously described [12]. To produce the nuclear-only ERα (NOER), which exhibits solely nuclear signaling [10], the C451A mutation was introduced into WT ERα using the QuikChange II Site-Directed Mutagenesis Kit (Agilent Technologies, Santa Clara, CA) and subsequently used to produce an adenoviral vector (Vector Biolabs, Philadelphia, PA), resulting in “Ad-NOER”. All ERα molecules were FLAG-epitope tagged at the N-terminus. hFOB cells were plated in 10-cm culture dishes (n = 6) at a final density of 2×10^4^ cells/cm^2^. Cells were infected at the time of plating at a multiplicity of infection (MOI) of 15 (Ad-ERα), 30 (Ad-NERKI) and 22.5 (Ad-NOER), which is herein shown to result in approximately equivalent protein levels for ERα, NERKI and NOER (Fig. 1). The cells were infected in the presence of 8 µg/mL hexadimethrine bromide (Sigma-Aldrich, St. Louis, MO) to enhance adenoviral infection. Following another 24 hour incubation to allow for complete infection, the cells were treated with either ethanol vehicle (0.1% v/v) or 10 nM 17-β-estradiol (0.1% v/v; Sigma-Aldrich) in the presence of media containing triple-stripped charcoal-treated FBS for an additional 24 hrs.

### Western Blotting

Whole cell extracts from adenoviral-infected hFOB cells was prepared and subjected to western blotting analysis as previously described [12]. A total of 10 µg protein, as determined by the Pierce BCA Protein Assay Kit (Thermo Scientific, Rockford, IL) was run from each adenoviral condition. The blots were incubated with primary antibodies directed against mouse-anti-Flag (1∶1000) and mouse-anti-β-actin (1∶10,000). The mouse anti-IgG secondary antibody linked to horseradish peroxidase was used at 1∶5000 dilution. All antibodies were purchased from Sigma-Aldrich. Blots were visualized using enhanced chemiluminscence (GE Lifesciences, Piscataway, NJ) and exposed to X-ray film.

### RNA/cDNA isolation

Total RNA was prepared using RNeasy minicolumns (Qiagen, Valencia, CA) and treated with RNase-free DNase (Qiagen) to remove potential contaminating DNA, as previously described [12]. The resulting RNA was used for either RNAseq analysis or in semi-quantitative real-time PCR analysis (QPCR). For the latter analysis, one microgram of total RNA was used in a reverse transcriptase (RT) reaction using the High Capacity cDNA Reverse Transcription Kit (Applied Biosystems by Life Technologies, Foster City, CA). The cDNA was diluted 1∶5 with water prior to QPCR analysis.

### RNA sequencing (RNAseq)

RNA libraries for RNAseq analysis were prepared from 100 ng of isolated total RNA from each sample using the manufacturer's instructions. Unique indexes were incorporated at the adaptor ligation step for loading multiple samples per flow cell. Three distinct indexed libraries were loaded per flow cell and sequenced on an Illumina HiSeq 2000 using TruSeq SBS sequencing software (version 3) and SCS data collection software (version 1.4.8). Base calling was performed using Illumina RTA (version 1.12.4.2). An average of 130 million reads per sample was achieved resulting in ∼96% mapped reads. A more detailed description of the RNAseq methodology used is as previously described [13]. The RNAseq data discussed in this publication have been deposited in NCBI's Gene Expression Omnibus [14] and are accessible through GEO Series accession number GSE55769 (http://www.ncbi.nlm.nih.gov/geo/query/acc.cgi?acc=GSE55769).

### Semi-quantitative Real-time PCR Analysis (QPCR)

The PCR reactions were run in the ABI Prism 7900HT Real-Time System (Applied Biosystems, Carlsbad, CA) using the Quantitect SYBR Green Master Mix (Qiagen), as previously described [15]. The method for data normalization using multiple reference genes and threshold calculations are as previously described [15]. Primer sequences for individual genes were designed using the Primer Express program (Applied Biosystems) and are available on request.

### Statistical analyses

For the QPCR analyses, calculations and statistical comparisons were performed using Microsoft Office Excel 2003 (Microsoft Corp., Redmond, WA) and the data are presented as the mean ± SEM. All values of p<0.05 were considered statistically significant using two-sided *t* tests. The RNAseq data was analyzed essentially as previously described [13]. Briefly, paired-end reads from the raw RNAseq data were aligned using TopHat (version 2.0.6) [16] against the h19 genome build using the bowtie 1 option [17], and quality control assessments were made using RSeQC software [18]. Gene counts were generated using HTSeq software and gene annotation files were obtained from Illumina. Gene expression data exhibiting a p-value≤0.05, a false discovery rate of q≤0.05 and median gene counts in at least one group of 10, were used for further investigation. In order to identify which genes are regulated via non-ERE or extra-nuclear actions of ERα, the WT ERα data was compared the NERKI and/or NOER RNAseq datasets. Overlapping genes were identified using the “Venny” web resource (http://bioinfogp.cnb.csic.es/tools/venny/). For the genes regulated via each of the three modes of action, pathway analysis was performed using the Ingenuity Pathway Analysis software (Ingenuity Systems, Redwood City, CA). Gene ontology (GO) terms from each dataset were generated using DAVID Bioinformatics Resources Version 6.7 [19].

## Results

### RNAseq analysis of hFOB human osteoblastic cells expressing ERα modal variants

The goal of this study was to identify and characterize estrogen-dependent gene expression patterns elicited by ERα through the nuclear ERE-dependent, nuclear ERE-independent, or through extra-nuclear signaling (e.g. membrane) in the ER-negative, human osteoblastic cell line hFOB [11] using global RNA sequencing (RNAseq). As tools to facilitate this approach, we utilized an ERα mutation that eliminates DNA binding through EREs (NERKI) [2], [20] and an ERα mutation which can only signal through the nucleus (NOER) [10]. By comparing the gene expression patterns of these mutant ERα receptor with wild-type ERα (WT), we can identify those patterns elicited by each mode of ERα action. Importantly, our analysis required that estrogen-regulated genes in each of the three modes of action were also regulated in WT, thus ensuring that these are physiologically regulated genes by ERα. Therefore, hFOB cells were infected with adenoviruses expressing FLAG-tagged WT, NERKI or NOER. Western blot analysis confirmed that all ERs were expressed at ∼equal levels (Figs. 1A/B).

To identify differentially expressed genes with each of the ERα modal variants, hFOB cells were infected with adenoviral vectors expressing WT, NERKI or NOER receptors, treated with estrogen (10 nM E2) for 24 h and RNAseq was performed (see Materials and Methods for details). In a previous study, we had determined that genes exhibiting a median gene count of at least 10 in one group represent an “expressed” gene [13]. Therefore genes with a median gene count of less than 10 in both comparison groups were called “non-expressed” and excluded from further analysis. In this study, the remaining genes exhibiting a p-value≤0.05 and a false discovery rate of q≤0.05 between control and estrogen treatments were used for further analyses.

Using these criteria, an estrogen-regulated gene list for WT was generated. In the WT treatment groups where all modes of estrogen action are preserved, a total of 4353 estrogen-regulated genes were identified (Table S1). Thirty randomly chosen genes from this dataset were analyzed by QPCR to validate the RNAseq data and a high correlation (r = 0.98, *P*<0.001) was observed (Figure S1), demonstrating a high level of accuracy of the RNAseq technology.

**Figure 2:**
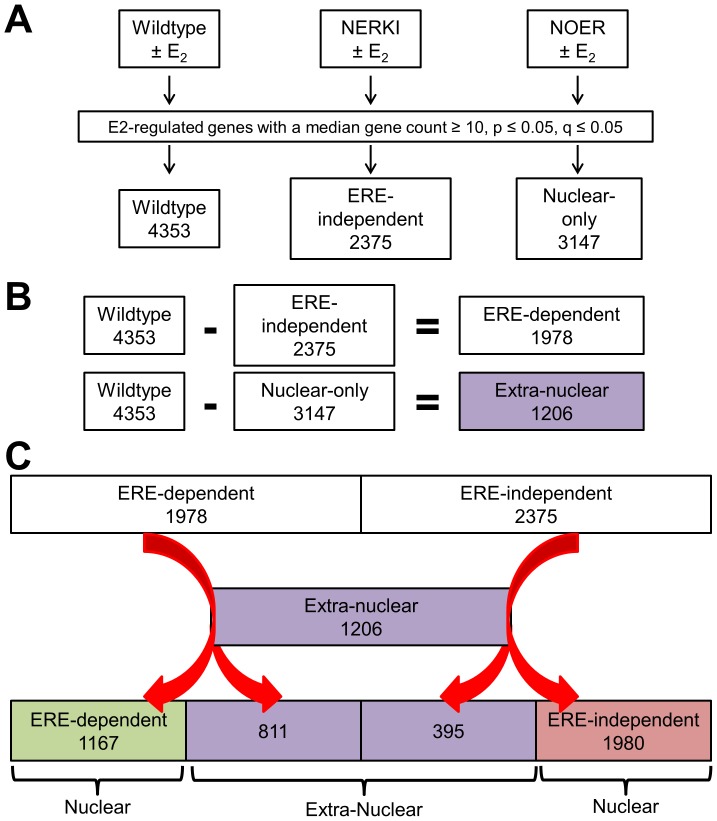
Flow-chart detailing our data analysis strategy (described in detail in the text).

**Figure 3:**
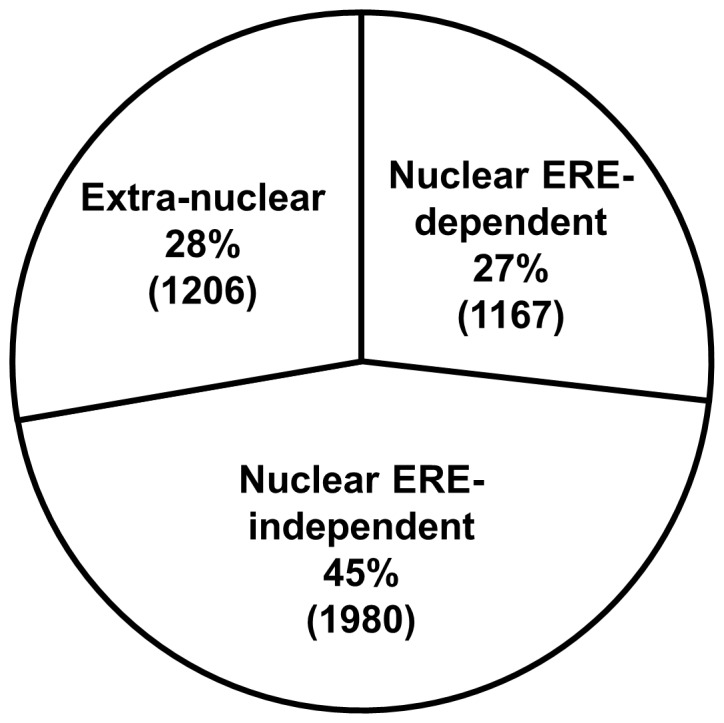
Confirmation of the RNAseq datasets and percentage breakdown of all estrogen-regulated genes among the three modes of ERα action. Breakdown of all the estrogen-regulated genes in the RNAseq analysis among the modes of ERα action, showing percentages and the number of genes from each mode in parentheses.

The flow-chart depicted in Figure 2 describes our data analysis strategy in detail. Similarly to WT, the NERKI and NOER datasets were passed through our data filters (median gene count≥10, p≤0.05 and q≤0.05) and 2375 estrogen-regulated genes were identified from the NERKI dataset (termed “ERE-independent”) and 3147 estrogen-regulated genes were identified from the NOER dataset (termed “Nuclear-only) (Fig. 2A). Complete lists for all three datasets can be found in Table S1 (tabs 1–3, respectively). To identify the “ERE-dependent” genes, the 2375 genes from the NERKI dataset were subtracted from the 4353 genes from the WT dataset (Fig. 2B; top row). This subtraction results in 1978 genes that are regulated by WT but not regulated by NERKI, therefore by inference these genes are regulated via ERE-dependent mechanisms. Similarly, to identify those genes whose estrogen-dependent regulation occurs from signals generated outside the nucleus (termed “Extra-nuclear), the 3147 genes in the Nuclear-only dataset were subtracted from the 4353 genes from the WT dataset (Fig. 2B, bottom row). This subtraction results in 1206 genes that are regulated by WT, but not the NOER, suggesting these genes are regulated in part by mechanisms originating outside of the nucleus. However, our ERE-dependent and ERE-independent gene lists include regulation occurring in both the nucleus and outside of the nucleus. To identify nuclear genes that are ERE-dependent and ERE-independent, each of these lists were overlaid with the 1206 genes in the Extra-nuclear list. For the ERE-dependent genes, this resulted in 1167 genes termed “Nuclear ERE-dependent” and with 811 genes from the Extra-nuclear list (Fig. 2C; left side). Similarly, for the ERE-independent genes, this resulted in 1980 genes termed “Nuclear ERE-independent” with 395 genes from the Extra-nuclear list (Fig. 2C; right side). Note that the sum of the Extra-nuclear genes from both comparisons is the complete Extra-nuclear gene list (811+395 = 1206). In this way, the lists comprising the three modes of ERα action (Nuclear ERE-dependent, Nuclear ERE-independent, Extra-nuclear) were identified. Complete lists for these datasets can be found in Table S2 (tabs 1–3, respectively). The percentage breakdown and number of estrogen regulated genes from each dataset representing these distinct modes of ERα action is shown in Figure 3. The top 20 up- and down-regulated genes for each dataset are shown in Tables 1–3.

**Table 1:** Most Highly Regulated Genes in the Nuclear ERE-dependent Dataset.

Up-regulated Genes			Down-regulated Genes		
Gene Symbol	WT	NOER	Gene Symbol	WT	NOER
*PGLYRP2*	103.00	5.56	*GALNT13*	0.22	0.32
*C11orf86*	25.39	13.59	*SLITRK1*	0.26	0.30
*MIR1286*	17.33	122.68	*KCNJ2*	0.27	0.41
*TH*	15.79	11.60	*C6orf141*	0.33	0.41
*RNF223*	15.72	7.38	*DUSP6*	0.36	0.33
*TMIE*	12.46	36.10	*TNF*	0.36	0.47
*NKX2-3*	10.27	2.72	*LINC00158*	0.36	0.31
*AQP5*	8.70	8.02	*CLDN14*	0.38	0.50
*IGLON5*	7.35	5.04	*C18orf1*	0.39	0.29
*OXT*	6.90	9.97	*KLF14*	0.42	0.47
*WISP2*	6.50	8.57	*MIR125B1*	0.43	0.23
*MYT1L*	6.37	7.11	*VIT*	0.43	0.63
*HS3ST6*	6.27	4.00	*LOC100506895*	0.44	0.51
*GFAP*	6.26	3.52	*IL6*	0.45	0.34
*FOXE3*	6.20	4.25	*GMNC*	0.48	0.57
*MIOX*	6.13	3.79	*ESM1*	0.50	0.57
*CDX2*	6.08	12.87	*SLITRK6*	0.51	0.47
*FGF8*	6.01	7.18	*ARL14*	0.51	0.55
*LOC389332*	5.08	7.58	*RUNX1T1*	0.51	0.70
*LOC728228*	4.93	3.97	*MYCT1*	0.52	0.45

**Table 2:** Most Highly Regulated Genes in the Nuclear ERE-independent Dataset.

Up-regulated Genes				Down-regulated Genes			
Gene Symbol	WT	NOER	NERKI	Gene Symbol	WT	NOER	NERKI
*ELF3*	26.61	17.94	2.66	*FGF20*	0.19	0.39	0.42
*LOC100507584*	24.54	14.55	2.67	*CCDC144NL*	0.21	0.18	0.38
*SMTNL2*	19.54	7.30	2.59	*HIF1A-AS2*	0.26	0.34	0.56
*VSX2*	12.87	4.78	5.49	*ADAMTS1*	0.29	0.29	0.41
*RASD1*	11.34	11.08	2.59	*LOC344887*	0.30	0.43	0.46
*CYTH4*	11.13	3.95	2.22	*RASSF9*	0.30	0.42	0.60
*GATA5*	11.10	9.15	3.82	*BHLHE22*	0.32	0.37	0.59
*LOC100127888*	10.91	16.22	3.38	*DKK1*	0.33	0.28	0.35
*CEBPA*	9.19	10.42	2.61	*PTGER4*	0.34	0.35	0.61
*NT5C1A*	8.80	5.19	3.16	*LURAP1L*	0.35	0.41	0.77
*LOXL4*	8.72	8.37	1.43	*CITED2*	0.36	0.33	0.45
*WFIKKN2*	8.51	6.38	1.83	*DNM3OS*	0.36	0.29	0.92
*PDGFB*	8.47	9.23	3.59	*MC4R*	0.36	0.51	0.57
*DLL4*	8.27	7.56	1.52	*ESR1*	0.39	0.47	0.70
*C16orf11*	8.06	7.29	1.47	*LOC730755*	0.40	0.47	0.63
*TAS1R1*	8.06	2.95	2.22	*MIR221*	0.40	0.36	0.45
*FGF4*	7.91	7.03	2.30	*SSTR1*	0.42	0.43	0.58
*GPR20*	7.70	6.24	6.68	*NFKBIZ*	0.43	0.46	0.58
*ALPPL2*	7.70	10.67	2.57	*HAS2*	0.43	0.41	0.64
*SOX18*	7.63	11.73	4.26	*RARG*	0.44	0.36	0.68

**Table 3:** Most Highly Regulated Genes in the Extra-nuclear Dataset.

Up-regulated Genes		Down-regulated Genes	
Gene Symbol	WT	Gene Symbol	WT
*WNT3A*	84.99	*ADCY2*	0.07
*UNCX*	45.10	*MIR483*	0.12
*LEFTY1*	15.34	*SLC32A1*	0.15
*POM121L9P*	10.56	*LINC00189*	0.17
*OTOP2*	10.33	*KRT84*	0.18
*KLK4*	7.42	*ZIC5*	0.23
*APOA4*	6.94	*ACSM4*	0.26
*PSAPL1*	6.03	*MIR1469*	0.29
*MYCN*	5.65	*HIST1H3A*	0.30
*TLX1*	5.59	*TCEAL2*	0.30
*CPN2*	5.23	*LOC100129046*	0.31
*PPP1R27*	5.21	*KRT5*	0.35
*LCN2*	5.15	*MEOX2*	0.35
*MYH6*	4.95	*GPR88*	0.37
*INSM1*	4.91	*ISL1*	0.37
*LOC727710*	4.81	*BLID*	0.39
*C1orf61*	4.73	*KCNJ2-AS1*	0.39
*MB*	4.70	*LOC401164*	0.39
*CHRND*	4.40	*RNU6ATAC*	0.40
*LOC100507218*	4.33	*SPINLW1*	0.41

### Ingenuity pathway analysis (IPA) and gene ontology (GO) analysis of the datasets from the three modes of ERα action

Following global identification of estrogen-regulated genes comprising the three modes of ERα action (described above), we performed IPA on each dataset to identify those cellular pathways mostly like regulated in each of these modes (see Table S3 for complete lists). We focused on those pathways with known regulatory roles in osteoblast biology. Pathways with known roles in bone biology for the Nuclear ERE-dependent (Table 4), Nuclear ERE-independent (Table 5) and Extra-nuclear (membrane) (Table 6) are indicated.

**Table 4:** Selected Pathways of Interest with Known Function in Bone in the “Nuclear ERE-dependent” Dataset.

Top Canonical Pathways	*P* Value	Ratio	Genes
GNRH Signaling	0.0081	0.11	*MAP3K9,SRC,MAP3K6,MAPK8,DNM3,DNM1, PAK3,CREB1,ITPR3,PRKAR1B,PRKCE,PRKCH,MAP2K3,ELK1,MAP2K1*
EGF Signaling	0.0166	0.14	*SRC,ITPR3,MAPK8,PIK3CD,CSNK2B,ELK1, MAP2K1,RASA1*
IL-8 Signaling	0.0240	0.09	*RAC2,SRC,PLD2,MAPK8,HBEGF,LIMK2,BAX, CSTB,EIF4EBP1,CCND3,PRKCE,PIK3CD, PRKCH,GNA13,KDR,RHOF,MAP2K1,IRAK2*
Sonic Hedgehog Signaling	0.0263	0.16	*ADRBK1,PTCH1,PRKAR1B,HHIP,DYRK1A*
VDR/RXR Activation	0.0380	0.12	*SPP1,CCNC,RUNX2,IL1RL1,MXD1,PRKCE, PRKCH,SEMA3B,CYP27B1*
PDGF Signaling	0.0380	0.11	*SRC,MAPK8,PDGFRA,PIK3CD,CSNK2B,ELK1, MAP2K1,RASA1,INPP5D*
p38 MAPK Signaling	0.0427	0.10	*TGFBR2,DAXX,ATF1,IL1RL1,MEF2D,CREB1, MEF2A,MAP2K3,ELK1,TNF,FAS,IRAK2*

**Table 5:** Selected Pathways of Interest with Known Function in Bone in the “Nuclear ERE-independent” Dataset.

Top Canonical Pathways	*P* Value	Ratio	Genes
Role of Osteoblasts, Osteoclasts and Chondrocytes in Rheumatoid Arthritis	0.0000	0.22	*BMP4, NFATC3, PIK3R1, TCF7, BMP8A, TGFB1, PIK3CG, WNT7B, WNT4, SMAD1, ADAMTS5, BMP8B, TNFRSF11B, WNT9A, PPP3CC, MAPK12, PIK3R3, IL18, SFRP1, RELA, NFKBIE, FZD1, NFKB1, NFATC1, SMURF1, JUN, NFKBIA, BMPR1A, NGFR, TNFRSF1B, TNFRSF1A, DVL1, DLX5, IKBKE, NFATC4, TNFRSF11A, CALM1, FZD8, FOS, TRAF2, FZD4, FOXO1, CSF1, IL1B, DKK1, BMP6, WNT11, LRP1, TCF7L2*
PPAR Signaling	0.0000	0.26	*PPARA,RELA,PDGFA,NFKBIE,NFKB1,NR2F1, NFKBIA,JUN,NGFR,MRAS,TNFRSF1B,CITED2, TAB1,TNFRSF11B,SRA1,TNFRSF1A,IKBKE, PDGFB,AIP,FOS,TRAF2,IL18,IL1B,NCOR2, PTGS2,RXRA*
Wnt/β-catenin Signaling	0.0000	0.22	*CDKN2A,TGFBR3,PPP2R5B,GSK3A,FZD1, RARG,CCND1,TCF7,JUN,NLK,TGFB1,WNT7B, RARA,TGFB2,WNT4,SOX18,TAB1,SOX4,SOX7, CSNK1G2,WNT9A,DVL1,SOX11,ACVR1B,FZD8, FZD4,TLE4,TLE3,SOX8,DKK1,PIN1,SFRP1, DVL2,ACVR2A,WNT11,LRP1,TCF7L2*
IL-6 Signaling	0.0000	0.23	*RELA,SOCS3,SOCS1,NFKBIE,PIK3R1,NFKB1, VEGFA,JUN,NFKBIA,NGFR,PIK3CG,MRAS, TNFRSF1B,MAPKAPK2,TAB1,TNFRSF11B, MCL1,IL8,TNFRSF1A,IKBKE,CEBPB,MAPK12, PIK3R3,FOS,IL18,TRAF2,IL1B,HSPB7*
TGF-β Signaling	0.0000	0.25	*RUNX3,BMP4,SMAD3,SKI,SMAD7,PITX2, MAPK12,ACVR1B,INHBB,INHBA,SMURF1,FOS, PIAS4,JUN,BMPR1A,TGFB1,MRAS,TGFB2, TFE3,SMAD1,ACVR2A,TAB1*
BMP signaling pathway	0.0003	0.23	*RELA,BMP4,FST,SMAD7,NFKB1,MAPK12, PITX2,NOG,SMURF1,JUN,BMP8A,BMPR1A, MRAS,PRKAG2,BMP6,SMAD1,BMP8B,TAB1*
VDR/RXR Activation	0.0005	0.23	*IL12A,PDGFA,HES1,CEBPB,THBD,HR,KLF4, GTF2B,GADD45A,FOXO1,PRKCD,HOXA10, CDKN1A,IGFBP3,CEBPA,TGFB2,NCOR2,RXRA*
NF-κB Signaling	0.0014	0.17	*RELA,BMP4,NFKBIE,TGFBR3,PIK3R1,NFKB1, NGF,FGFR3,NFKBIA,CARD10,BMPR1A,NGF, PIK3CG,NTRK1,MRAS,TNFRSF1B,TAB1, TNFRSF11B,TNFRSF1A,RELB,MALT1, TNFRSF11A,TLR9,PIK3R3,TLR4,IL18,TRAF2, TGFA,IL1B*
Notch Signaling	0.0028	0.24	*DLL1,JAG2,MFNG,LFNG,HES1,JAG1,DLL4, NOTCH1,DTX2,HEY1*
IGF-1 Signaling	0.0032	0.19	*SOCS1,SOCS3,IGFBP4,CTGF,JAK1,PIK3R1, SOCS2,SOCS6,PIK3R3,FOS,JUN,FOXO1, PIK3CG,IRS1,IGFBP3,MRAS,PRKAG2,IRS2, CYR61*
Estrogen Receptor Signaling	0.0457	0.14	*MED12L,PELP1,SRA1,TAF6,PCK1,TAF10, ERCC2MED12,G6PC3,TAF6L,GTF2B,POLR2A, MED17,GTF2H4,GTF2E1,MRAS,MED16,NCOR2, ESR1*

**Table 6:** Selected Pathways of Interest with Known Function in Bone in the “Extra-nuclear” Dataset.

Top Canonical Pathways	*P* Value	Ratio	Genes
Integrin Signaling	0.0014	0.11	*RAP1B,PIK3CA,TSPAN5,MPRIP,DIRAS3,ACTA2,ABL1,PIK3R5,TSPAN2,RHOJ,PIK3R4,PTEN, MYL9,ITGAE,ITGA3,PAK1,CAPNS1,GRB7, RHOU,ACTC1,ITGB5,CAPN10*
mTOR Signaling	0.0014	0.11	*NAPEPLD,PIK3CA,PLD3,PRKAB1,DIRAS3, PIK3R5,PPP2R3B,RPS8,VEGFB,RHOJ,PIK3R4, RICTOR,EIF4G1,RPS7,PRKAA2,EIF3A,RHOU, PRR5,PPP2R2C,INSR,RPS14*
FAK Signaling	0.0087	0.11	*PIK3CA,ITGA3,PAK1,CAPNS1,ACTA2,PIK3R5, PIK3R4,TNS1,ACTC1,CAPN10,PTEN*
AMPK Signaling	0.0148	0.10	*PIK3CA,PRKAB1,PIK3R5,PPP2R3B,LIPE,PIK3R,ADRB1,FASN,PRKAA2,PPP2R2C,CPT1C, MLYCD,INSR,CHRNA3*
Calcium Signaling	0.0155	0.09	*RAP1B,MYH6,ACTA2,HDAC10,TPM3,MYH7, CHRND,MYH7B,GRIA4,MYL9,CAMK2A,MYH2, CASQ1,ASPH,ACTC1,CHRNA3,CAMK2G*
p53 Signaling	0.0363	0.11	*PIK3CA,THBS1,E2F1,PIK3R5,C12orf5,MDM2, PIDD,PIK3R4,PTEN,TP53I3*
eNOS Signaling	0.0468	0.09	*BDKRB2,NOSIP,ADCY2,PIK3CA,CCNA1, HSPA14,PRKAB1,PRKAA2,PIK3R5,VEGFB, PIK3R4,CHRNA3*
TR/RXR Activation	0.0490	0.10	*SLC16A3,PIK3CA,ADRB1,COL6A3,FASN, PIK3R5,NCOA4,MDM2,PIK3R4*

We also performed gene ontology (GO) analysis to categorize the target genes regulated in each mode of ERα action into specific molecular functions (GOTERM_MF_FAT) or cellular components (GOTERM_CC_FAT) (see Table S4). We found that the target genes regulated in the nuclear ERE and non-ERE mode of ERα action were largely enriched for genes involved in transcriptional regulation. Interestingly, the target genes regulated by the extra-nuclear mode of ERα action, presumably occurring through membrane-associated processes, were largely enriched for genes involved in metabolic pathways (i.e. NADH/NADPH oxidoreductase activity and FAD binding) and structural components of the ribosome.

### Comparison of the datasets representing the three modes of ERα action with genes with known involvement in bone biology

**Figure 4:**
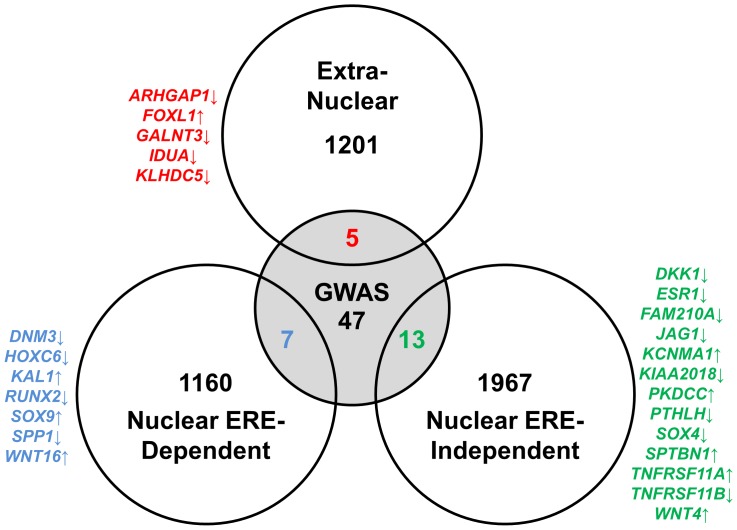
Comparison of the RNAseq datasets to gene with known involvement in bone biology from genome-wide association studies (GWAS). The nuclear ERE-dependent, nuclear ERE-independent, and extra-nuclear datasets were intersected with the 72 genes identified by GWAS studies. The overlapped genes for each mode of ERα action are listed and color-coded for clarity. The directionality of the estrogen-dependent regulation is denoted with an arrow following the gene symbol.

To place our results in a more physiologically relevant context in terms of bone biology, we intersected our gene lists corresponding to the three modes of ERα action with a recent meta-analysis that identified 72 genomic loci with genome-wide significance to bone mineral density and/or fracture rates [21]. As seen in Figure 4, a total of 25 (∼35%) of the GWAS-identified genes are found in our datasets.

## Discussion

Estrogen receptor (ER)-α can utilize a number of different mechanisms to modulate gene expression, including nuclear ERE-dependent, nuclear ERE-independent, and extra-nuclear signaling (e.g. membrane). The goal of this study was to identify the global gene expression patterns regulated by each of these mechanisms in the ER-negative, human osteoblastic cell line hFOB [11] using global RNA sequencing (RNAseq), in an effort to further understand how each mode of ERα action contributes to the overall response of osteoblasts to estrogen. Using specific ERα mutants which eliminate one mode of ERα action, and by comparing these datasets to the WT dataset, we were successful in identifying genes regulated by either the nuclear ERE-dependent, nuclear ERE-independent, and extra-nuclear actions of ERα.

Examination of the relative proportions of the modes of ERα action revealed that a majority of estrogen-regulated genes (72%) are regulated at least partially via nuclear mechanisms. This data is supported by recent report that 82% of estrogen-regulated genes in the uterus are regulated via nuclear ERα pathways [22]. Of these, nearly two-thirds (63%) are from ERE-independent mechanistic pathways and one-third (37%) from ERE-dependent mechanistic pathways. This suggests that a common mechanism for ERα to influence estrogen-dependent transcription in the nucleus is through cooperation with other DNA-bound transcription factor complexes. Our pathway analysis of each mode of ERα action supports this contention, since 135 cellular pathways were significantly regulated by the nuclear ERE-independent dataset, whereas only 48 and 33 pathways were significantly regulated in the nuclear ERE-dependent and extra-nuclear datasets, respectively.

Recent research has demonstrated that ERα is found at the plasma membrane of nearly all ERα-expressing cells [10], and therefore contributes to overall estrogen signaling in most ER-positive cells, including osteoblasts. Indeed, we find that 28% of all estrogen-responsive genes originate from extra-nuclear (e.g. membrane) compartment, since they are regulated by WT ERα but not the nuclear-only ERα (e.g. NOER). In addition, accumulating evidence now suggests that significant cross-talk occurs between the membrane and nuclear ERα signaling [10], [23], [24], [25]. A well-known example of this cross-talk is in the estrogen-dependent regulation of the *Ccnd1* gene in breast cancer cells, where membrane-initiated activation of PI3K/AKT and ERK signaling, as well as ERE-dependent mechanisms are necessary for maximal estrogen-stimulated transcription [7], [26], [27].

An important and interesting aspect of this research is that estrogen-regulated pathways that are either in common or unique to the three modes of ERα action can be identified. Comparison of the pathways from all modes of ERα action revealed a few shared pathways, such as: integrin and integrin linked kinase (ILK) signaling, tight junction signaling, IL8 signaling, MAPK mTOR signaling. These may represent a more generalized function of ERα action, in which multiple modes are involved. However, most of the significant pathways are unique to each mode, suggesting a unique biology is involved dependent on how ERα is functioning. Unique pathways regulated in the nuclear ERE-dependent mode include EGF signaling, sonic hedgehog signaling and many cholesterol and nucleotide biosynthetic pathways. This is in contrast to the more common signaling pathways regulated in the nuclear ERE-independent mode of ERα action such as PPAR, BMP, Wnt, GR, IL6, TGFβ, among many others. Unique signaling pathways in the extra-nuclear mode of ERα action include FAK, AMPK, calcium and eNOS signaling. Understanding how each mode regulates a unique aspect of overall ERα signaling in osteoblasts may lead to the generation of “mode-specific” ligands used to target specific pathways in estrogen-responsive cells.

At a physiological level, estrogen signaling is well recognized as an important determinant of bone mineral density (BMD), as declining estrogen levels during the menopause lead to increased bone turnover and bone loss, as is observed in postmenopausal osteoporosis [1]. Therefore it is not surprising that significant overlap exists upon intersection of our estrogen-dependent gene lists with the 72 loci identified in GWAS studies as being related to BMD and/or fracture rates [21]. These include genes with well-known roles in osteoblast biology, such as *Runx2*, *Spp1* (*osteopontin*), *Esr1 (ERα)* and *Tnfrsf11b (Opg)*, as well as other genes with less recognized roles in bone. This demonstrates that all modes of ERα action contribute to gene regulatory patterns important in bone biology. Future studies are needed to examine the role of these genes in bone diseases such as osteoporosis.

In reality, these modes of ERα action do not exist in isolation. It is well known that the rapid effects of membrane estrogen signaling have modulatory effects on nuclear estrogen signaling through phosphorylation of ERα and/or coregulatory transcription factors, as well as induction of numerous signaling cascades (reviewed in [6], [28]). In this way, elimination or modulation of any one mode of ERα action affects the whole system, eventually affecting gene expression and phenotypic expression of the cell, and ultimately the organ and organism [9]. These actions may be very different in various estrogen-responsive tissues, depending on the amount of ERα present and the complement of transcription factors expressed in that particular cell.

Since we treated the ER-infected hFOB cells for 24 h, our data most certainly contains not only genes that directly respond to ligand-bound ERα (a primary response), but also those genes whose regulation is dependent on primary response genes (e.g. secondary response genes). Although this is an important aspect to consider when examining the gene lists produced in this study, our primary premise is that these 3 modes of ERα action which are mechanistically distinct, ultimately put into motion a mode-specific program that leads to the regulation of distinct subsets of genes. Future studies with shorter treatment periods will be necessary to limit these lists to primary response genes.

We recognize that our study has several limitations. First, we are using an *in vitro* cell culture system and since the hFOB cells are ER-negative, we expressed these ER mutations using an adenoviral vector. Using an ER-negative cell model was essential to eliminate any endogenous ER expression that could confound the results. In addition, during development of this cell culture system we carefully titrated the various adenoviral vectors and used the minimal amount to achieve equal expression of each receptor in >95% of the cells. Further, while we have not formally compared the levels of ERα expression in our transfected cells with a panel of WT osteoblasts, it is likely that these levels are considerably higher in our transfected cells, despite the fact that we tried to use fairly low viral titers for the transfections. This issue is compounded by the fact that levels of ERα expression *in vitro* vary considerably with the stage of osteoblast differentiation. Thus, while the strength of our *in vitro* system is that we were able to study the various estrogen signaling pathways in isolation, we recognize that further validation of these findings in *in vivo* systems with physiological levels of ERα are needed. A second limitation is that by using hFOB cells, we limited our study to osteoblasts. However, these datasets may be useful in identifying similar ERα action in other estrogen-responsive tissues, such as breast and uterus. Further studies are needed to examine the genes/pathways regulated by these modes of ERα action in other estrogen-responsive tissues. A third limitation is that only ERα, and not ERβ was studied. These modes of action also exist for ERβ, including the membrane-associated ERβ mechanistic pathway that has been extensively studied in cardiomyocytes [29], [30]. Future studies will be needed to clarify these modes of ERβ action and interaction with ERα, which will be considerably more complex.

In conclusion, we have identified three molecular modes of ERα action in human osteoblastic cells using RNAseq methodology. The identification of estrogen-dependent gene expression patterns and pathways within these modes of action is an important first step into understanding the complete picture of how ERα action contributes to overall ERα signaling in bone, and may provide the data necessary for generation of “mode-specific” ligands to preferentially influence a single mode of ERα action.

## Supporting Information

Figure S1 **QPCR confirmation of the RNAseq dataset from wild-type ERα.** Thirty randomly chosen genes from the wild-type ERα dataset were chosen and QPCR was performed on independent samples. The plot shows a high degree of concordance between the RNAseq dataset and the QPCR analysis, with an r = 0.98 and *P*<0.001.
(TIF)

Table S1 Complete gene lists of the estrogen-regulated genes from the 3 ERα variants in this study. The estrogen-regulated genes from the wildtype ERα, ERE-dependent, and Nuclear-only ERα variants (from Fig. 2A) are listed in each respective tab. The gene name, accession numbers, fold-change with estrogen (FC), p-value and false discovery rate (FDR) are indicated.
(XLSX)

Table S2 Complete gene lists of the estrogen-regulated genes comprising the 3 modes of ERα action. The “Nuclear ERE-dependent”, “Nuclear ERE-independent” and “Extra-nuclear” estrogen regulated genes are indicated (from Fig. 2C) with the same values as described in Table S1.
(XLSX)

Table S3 Complete Ingenuity pathway analysis (IPA) of the genes from the 3 modes of ERα action. The IPA pathways for the genes for each mode of ERα action are indicated.
(XLSX)

Table S4 Gene ontology (GO) analysis of the genes from the 3 modes of ERα action. Gene ontology (GO) analysis was performed to categorize the target genes regulated in each mode of ERα action into specific molecular functions (GOTERM_MF_FAT) or cellular components (GOTERM_CC_FAT).
(XLSX)
