# Supplementary figures and images for: Dissection of Estrogen Receptor Alpha Signaling Pathways in Osteoblasts Using RNA-Sequencing
Source: PLoS One. 2014 Apr 28;9(4):e95987. doi: 10.1371/journal.pone.0095987 (PMC4002480; doi:10.1371/journal.pone.0095987)

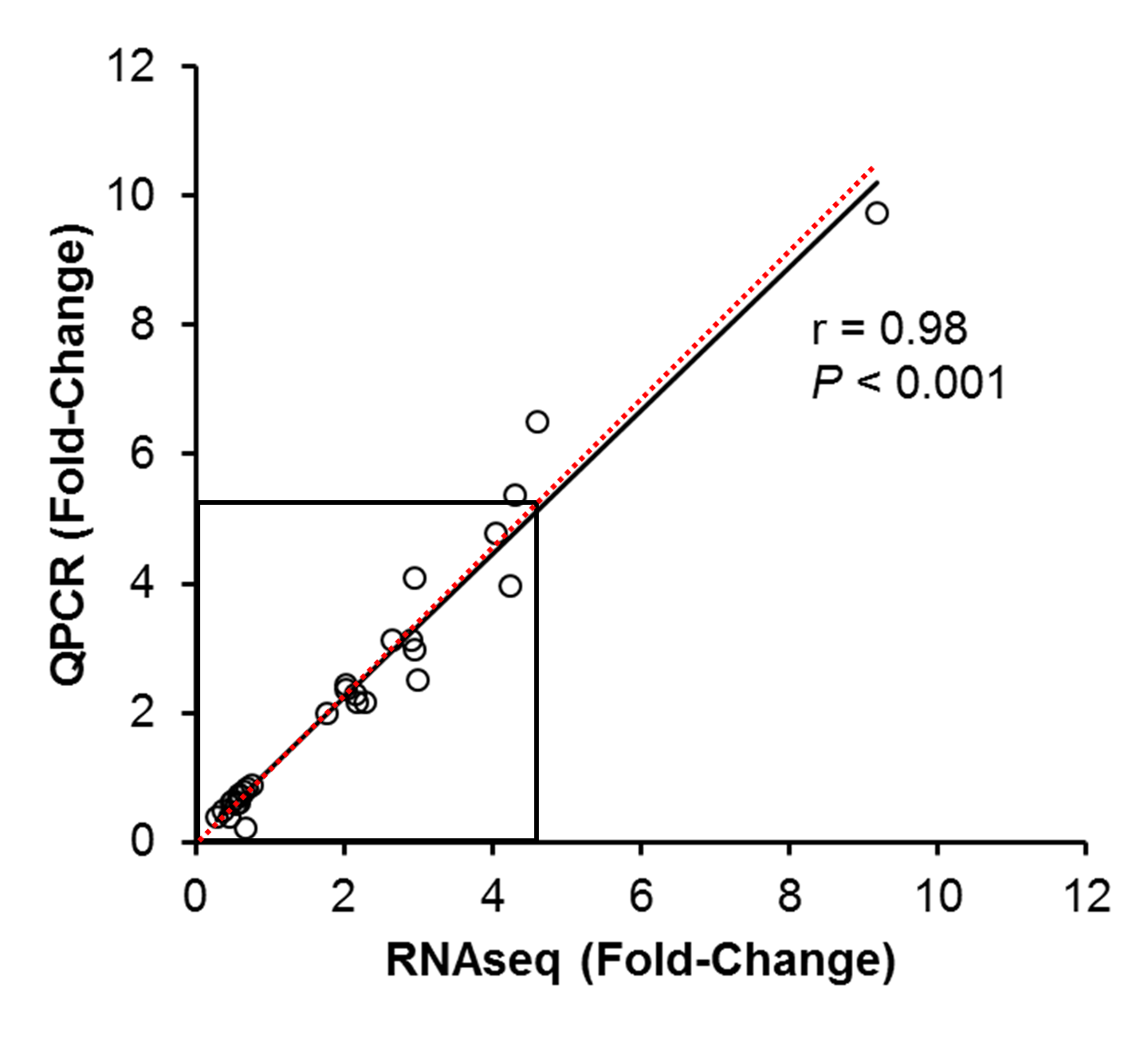

Supplement: Figure S1 — QPCR confirmation of the RNAseq dataset from wild-type ERα. Thirty randomly chosen genes from the wild-type ERα dataset were chosen and QPCR was performed on independent samples. The plot shows a high degree of concordance between the RNAseq dataset and the QPCR analysis, with an r = 0.98 and P<0.001. (TIF) [file pone.0095987.s001.tif]
